# The value of compassionate support to address smoking: A qualitative study with people who experience severe mental illness

**DOI:** 10.3389/fpsyt.2022.868032

**Published:** 2022-10-06

**Authors:** Kristen McCarter, Melissa L. McKinlay, Nadine Cocks, Catherine Brasier, Laura Hayes, Amanda L. Baker, David Castle, Ron Borland, Billie Bonevski, Catherine Segan, Peter J. Kelly, Alyna Turner, Jill Williams, John Attia, Rohan Sweeney, Sacha Filia, Donita Baird, Lisa Brophy

**Affiliations:** ^1^School of Psychological Sciences, College of Engineering, Science and Environment, University of Newcastle, Callaghan, NSW, Australia; ^2^Department of Mental Health, St Vincent's Hospital Melbourne, Fitzroy, VIC, Australia; ^3^Research, Advocacy and Practice Development, Mind Australia, Heidelberg, VIC, Australia; ^4^School of Allied Health, Human Services and Sport, La Trobe University Melbourne, Melbourne, VIC, Australia; ^5^School of Medicine and Public Health, Faculty of Health and Medicine, University of Newcastle, Callaghan, NSW, Australia; ^6^Centre for Complex Interventions, Centre for Addiction and Mental Health, Department of Psychiatry, University of Toronto, Toronto, ON, Canada; ^7^Melbourne School of Psychological Sciences, The University of Melbourne, Melbourne, VIC, Australia; ^8^Flinders Health and Medical Research Institute, College of Medicine and Public Health, Flinders University, Bedford Park, SA, Australia; ^9^Melbourne School of Population and Global Health, The University of Melbourne, Melbourne, VIC, Australia; ^10^Cancer Council Victoria, Melbourne, VIC, Australia; ^11^Illawarra Health and Medical Research Institute and the School of Psychology, University of Wollongong, Wollongong, NSW, Australia; ^12^IMPACT Strategic Research Centre, School of Medicine, Barwon Health, Deakin University, Geelong, VIC, Australia; ^13^Division of Addiction Psychiatry, Rutgers Robert Wood Johnson Medical School, New Brunswick, NJ, United States; ^14^Centre for Health Economics, Monash Business School, Monash University, Melbourne, VIC, Australia

**Keywords:** tobacco treatment, quitline, peer worker, mental illness, severe mental illness, health disparities

## Abstract

**Introduction:**

People experiencing severe mental illness (SMI) smoke at much higher rates than the general population and require additional support. Engagement with existing evidence-based interventions such as quitlines and nicotine replacement therapy (NRT) may be improved by mental health peer worker involvement and tailored support. This paper reports on a qualitative study nested within a peer researcher-facilitated tobacco treatment trial that included brief advice plus, for those in the intervention group, tailored quitline callback counseling and combination NRT. It contextualizes participant life experience and reflection on trial participation and offers insights for future interventions.

**Methods:**

Qualitative semi-structured interviews were conducted with 29 participants in a randomized controlled trial (intervention group *n* = 15, control group *n* = 14) following their 2-month (post-recruitment) follow-up assessments, which marked the end of the “Quitlink” intervention for those in the intervention group. Interviews explored the experience of getting help to address smoking (before and during the trial), perceptions of main trial components including assistance from peer researchers and tailored quitline counseling, the role of NRT, and other support received. A general inductive approach to analysis was applied.

**Results:**

We identified four main themes: (1) the long and complex journey of quitting smoking in the context of disrupted lives; (2) factors affecting quitting (desire to quit, psychological and social barriers, and facilitators and reasons for quitting); (3) the perceived benefits of a tailored approach for people with mental ill-health including the invitation to quit and practical resources; and (4) the importance of compassionate delivery of support, beginning with the peer researchers and extended by quitline counselors for intervention participants. Subthemes were identified within each of these overarching main themes.

**Discussion:**

The findings underscore the enormity of the challenges that our targeted population face and the considerations needed for providing tobacco treatment to people who experience SMI. The data suggest that a tailored tobacco treatment intervention has the potential to assist people on a journey to quitting, and that compassionate support encapsulating a recovery-oriented approach is highly valued.

**Clinical trial registration:**

The Quitlink trial was registered with ANZCTR (www.anzctr.org.au): ACTRN12619000244101 prior to the accrual of the first participant and updated regularly as per registry guidelines.

## Introduction

Severe mental illness (SMI) refers to a mental illness that results in serious functional impairment, which substantially interferes with or limits one or more major life activities (1, 2). People who experience severe mental illness (SMI) smoke tobacco at much higher rates than the general population and die on average 10–20 years earlier (3). Most of this mortality gap is attributed to smoking-related diseases such as cardiovascular disease, respiratory disease, and cancer (4). Smoking is therefore one of the major modifiable risk factors for premature mortality in this population. Evidence suggests that people who experience SMI are as motivated to quit as the general population (5). However, they have lower overall success with cessation (6, 7), which has been attributed to a range of factors including not routinely receiving tobacco treatment (8–10).

Gold standard interventions for tobacco treatment encompass pharmacotherapy (e.g., combination nicotine replacement therapy (NRT), varenicline, bupropion) coupled with multi-session behavioral counseling (11). Telephone delivery (i.e., a quitline) of tobacco treatment counseling has the potential to improve access and is beneficial for people who experience SMI (12–15). However, quitline services are underutilized (16) and more effort is required to enable engagement for people experiencing mental ill-health. Similarly, despite evidence for the effectiveness of pharmacotherapy for cessation for people who experience SMI (17–19), NRT is also underutilized in this population (20). To try to enhance uptake of both quitline and pharmacotherapy, we developed, in collaboration with the Victorian (Australia) Quitline, a tailored intervention for smokers who experience SMI that aims to address common cessation issues for people experiencing SMI, e.g., concerns that stopping smoking might worsen mental health, coping with more severe withdrawal symptoms due to higher levels of nicotine dependence, managing potential increases in medication side-effects and the upfront costs of combination NRT. The “Quitlink” quitline counseling intervention included structured monitoring of mental health symptoms, nicotine withdrawal symptoms, and medication side-effects (21), a dedicated Quitline counselor plus 8 weeks of free combination NRT.

Mental health peer workers can play a role in enhancing tobacco treatment interventions for people who experience SMI. Peer workers bring their lived experience of mental ill-health and recovery to engage and support consumers of mental health services. Recovery can be conceptualized as a process of building a meaningful and satisfying life, as defined by the person themselves, whether or not they are experiencing ongoing or recurring symptoms or problems associated with illness (22). Principles of recovery-oriented practice include openness, collaboration as equals, a focus on the individual's inner resources, reciprocity, and a willingness “to go the extra mile” (23). The growing recognition of the value that lived experience expertise can provide, along with recovery-oriented practice (24) means that peer workers have a significant role to play in supporting and empowering people who experience SMI to engage with tobacco treatment. Marginalization and stigma can play a role in reinforcing smoking behaviors and peer workers offer hope and connection (25).

People who experience SMI and smoke encounter complex barriers to tobacco use recovery, including higher levels of nicotine dependence and the potential for more severe withdrawal symptoms, greater likelihood of living with smokers, increased financial stress as well as stigma (including internalized stigma), social exclusion as well as the impact of mental illness and treatment (25). To evaluate tobacco treatment trials, consideration for the participant context and experience is necessary. Qualitative methodology can illuminate participant trial experience and engagement and provide a nuanced understanding of participants' broader context. The aim of the present study was to explore the experiences of participants in the “Quitlink” trial, particularly regarding quitting smoking and tobacco treatment.

## Methods

This study is reported according to the consolidated criteria for reporting qualitative studies (COREQ): 32-item checklist (26). Ethical approval was granted by St Vincent's Hospital, Melbourne (HREC Reference Number: HREC/18/SVHM/154), the University of Newcastle HREC (HREC Reference Number: H-2018-0192) and the Cancer Council Victoria, HREC (HREC Reference Number: 1807).

This qualitative study was nested within the “Quitlink” trial (27). Depending on recruitment strategy, participants were “invited” to the “Quitlink” trial *via* different methods including peer researcher presentation at mental health services, clinician referral, direct mail postcard, and Facebook advertising. The active control condition included advice to quit, encouragement to use NRT, and a Quit Victoria pack of written materials to motivate a quit attempt and encourage use of the Quitline service. The Quitlink intervention participants received the above and additionally, a proactive referral to Quitline counseling tailored to meet the needs of people experiencing SMI and up to 8 weeks of combination NRT. This tailored counseling was based on cognitive behavioral therapy and included structured monitoring of mental health symptoms, nicotine withdrawal symptoms, and medication side-effects to help distinguish temporary withdrawal symptoms from psychiatric symptoms; and a focus on psychoeducation including the relationship between smoking and mood; goal setting; identification of triggers to smoke; and facilitating problem solving and skills building, including the use of mood management strategies that also act to aid cessation (27).

For the current study, participants were recruited from both control and intervention arms of the trial.

### Sample

Eligible participants were those who participated in the “Quitlink” trial and provided consent to be contacted about participating in qualitative interviews. To be eligible for the main trial, participants smoked at least 10 cigarettes a day and were accessing treatment or support from participating mental health agencies. The majority of people accessing these services will have been experiencing SMI. As a result of slow recruitment and COVID-19, eligibility criteria were expanded during the trial to include people accessing support or treatment from their general practitioner, for a mental health or alcohol and other drug use condition. The Mini International Neuropsychiatric Interview [MINI; (28)] was administered at follow-ups to obtain lifetime mental health diagnosis. The McLean Screening Instrument for Borderline Personality Disorder (29) was administered to verify participant self-reported main diagnosis. Diagnoses were grouped into “psychotic” and “non-psychotic” disorders.

Peer workers were employed as peer researchers to help recruit participants, conduct baseline assessments, deliver brief advice and facilitate engagement with the study (27). Participants were randomized to either an active control condition or the Quitlink intervention and were blinded as to which group they were in. The quantitative component of the trial included subsequent follow-ups at 2-, 5-, and 8-months post-baseline. The focus of this paper is the qualitative interviews conducted soon after participants had completed 2-month follow-up (which marked the end of the “Quitlink” intervention period for those in the intervention group). Participants were also invited to further follow-up qualitative interviews to enable them to share their experiences over time: these results will be presented elsewhere.

To achieve variability, a decision was made to wait until a proportion of participants had completed their 2-month quantitative follow-up assessments before selecting and contacting participants. However, due to slower than expected and lower recruitment to the main trial, the intended purposive sampling strategy for the current study was replaced by convenience sampling. Subsequent delays in commencing the qualitative part of the study meant the first 25 people who had agreed to be contacted about an interview were already at the 5- or 8- month follow-up stage and were therefore not contacted to participate in the 2-month qualitative interviews. No participants who were recruited to the main trial *via* the initial recruitment strategy of face to face, peer researcher presentation participated in the 2-month qualitative interviews. Thus, they were invited to be included in 5- and 8-month qualitative interviews (these data will be presented in future papers).

### Procedures

Following completion of the 2- month follow up assessment, participants were interviewed about their experiences of being in the study to that point in time. Recruitment and interviewing were undertaken by a team of female researchers including the peer researchers (NC, MM, and CB) and the trial coordinator (KM). NC, MM, and CB (PhD) are peer researchers with a range of experience, and NC and MM recruited and delivered brief advice to participants as part of the main trial. KM (PhD) is a clinical psychologist and had previous experience leading qualitative research. Written or verbal recorded consent was obtained.

In order to facilitate recruitment and engagement and provide access to peer support for the main trial, peer researchers NC and MM were embedded within the mental health services where participants were recruited. The interviewers were supervised by LB (a senior investigator of the project with a social work background). Participants were aware that those conducting the interviews were a part of the main research trial team. Participants had previously interacted with the peer researchers and were aware that they were bringing their lived experience including their experience with mental ill-health and recovery and smoking to the study. However, peer researchers did not interview any of the participants they specifically recruited to the main trial.

Interviews were conducted *via* telephone with only the interviewer and interviewee present. A semi-structured interview guide was used (see Supplementary material). The interview guide was developed by LB in collaboration with investigators from the main trial and explored:

- Participant history of getting help to address smoking and current experience
- Perceptions of assistance from peer researchers to address smoking
- Impressions of Quitline counseling
- The role of NRT in participants' quit attempts
- Support received from other services, health professionals or support workers

The interviews were audio recorded and transcribed verbatim. Participants received an AU$40 gift card as renumeration for participation.

### Data analysis

Thematic analysis, employing a general inductive approach was applied (28, 29), identifying themes and patterns within the data, with no preconceived theories applied. While a general inductive approach is aligned with grounded theory, the underlying assumptions of a general inductive approach include that data analysis relates to both the research objectives and interpretation of raw data (30). To begin, KM conducted open coding of the first half of transcripts independently of the other authors, using NVivo 12. A series of team meetings were then held in which four transcripts were open coded by the other coders to identify, review, and refine the codes. Axial coding was then performed by KM to draw connections between codes, and categories were created.

Categories and codes were organized in a word document to assist in gaining a clearer sense of the data. From here, preliminary themes were identified by the team. This process allowed for discussion and deepened the interpretation of meaning within the data and aimed to increase the trustworthiness of the findings (30). Again, a series of weekly team meetings were conducted to review, confirm, and refine the themes and subthemes. We used a consensus approach to agree on themes and sometimes this led to lengthy discussions. The team were able to benefit from the input of the lived experience expertise of the peer researchers in the analysis. A summary of preliminary themes and illustrative quotes from the interviews was sent to participants (who had previously consented n =19) for their feedback.

## Results

For the current study, 29 interviews were conducted. Participant information can be found in Table 1. Participants in the qualitative study had a similar profile to the trial sample overall (31). Most of the participants in the current study were recruited to the main trial *via* direct mail postcard, followed by online advertising. The average age of participants was 46 years old and over half (59%) were female. Most participants met criteria for a psychotic disorder [MINI; (32)], the average number of cigarettes per day at recruitment was 20 and most participants scored in the moderate range for tobacco dependence as measured by the Heaviness of Smoking Index (33). Participants represented both intervention (*n* = 15) and control (*n* = 14) arms of the trial and a range of tobacco use recovery outcomes at their 2-month quantitative follow-up assessment (including cut down, quit, relapsed and continued smoking). Engagement with Quitline (from recruitment to 2-month quantitative follow-up assessment) ranged from 2 to 11 calls for intervention participants. Two of the control participants contacted Quitline independently following the brief advice provided to all participants at baseline (and received either one or four calls).

**Table 1:** Participant characteristics (collected in Quitlink trial baseline assessments) (*N* = 29).

**Characteristic**	***n* (%)**	**Mean (range)**
**Age**		46 (20–64)
**Female**	17 (59%)	
**Marital status**
Married/defacto/widowed	3 (10%)	
Separated/divorced	9 (31%)	
Single/never married	15 (52%)	
Other	2 (7%)	
**Education**		
Primary school/years 7–9	2 (6%)	
School certificate/intermediate/year 10/4th form	7 (24%)	
HSC/leaving/year 12/6th form	2 (7%)	
TAFE certificate/diploma/trade certificate or apprenticeship	12 (41%)	
University/College of advanced education/Some other tertiary institute degree or higher	6 (21%)	
**Employment status**		
No job	16 (55%)	
Housework/stay at home parent	1 (3%)	
Studying	2 (7%)	
Volunteer	1 (3%)	
Part time/casual/temporary worker	7 (24%)	
Full time	2 (7%)	
Diagnosis*(mini international neuropsychiatric interview)		
Psychotic disorders (schizophrenia spectrum disorders, bipolar 1 disorder, bipolar 1 disorder with psychotic features, or major depressive disorder with psychotic features)	20 (69%)	
Non-psychotic disorders (major depressive disorder, post-traumatic stress disorder, generalized anxiety disorder, borderline personality disorder)	7 (24%)	
Inconclusive	2 (7%)	
**Cigarettes per day at recruitment**		20 (11–35)
**Nicotine dependence**		
Low	1 (3%)	
Moderate	22 (76%)	
High	6 (21%)	
**Recruitment type**		
Face to face	0	
Postcard	24 (83%)	
Online	5 (17%)	
**Allocation**		
Intervention	15 (52%)	
Control	14 (48%)	
**Time between recruitment and interview (days)**		107 (65–145)
**Time between quantitative 2-month follow-up and interview (days)**		48 (18–84)

^*^Assessed at Quitlink trial follow-up assessments.

Defacto, partners in a relationship who live together as a couple but are not married; HSC, Higher School Certificate; TAFE, Technical and Further Education.

We identified four main themes, reflecting (1) the long and complex journey that illustrated the quitting histories of participants; (2) their considerations of the factors that affect quitting smoking; (3) the perceived benefits of a tailored approach for people experiencing mental ill-health; and (4) the importance of compassionate delivery of support. Subthemes were identified within each of these overarching main themes.

**Theme 1: A long and complex journey**

The interviews provided an understanding of how smoking is contextualized within participants' lives. Most described a long and complex journey with smoking and tobacco use recovery. Their comments highlighted the deeply intertwined intersection between smoking, challenging and disrupted lives and histories of multiple attempts to quit.

***Subtheme: Disrupted lives***

Participants reported disrupted and unpredictable lives. They spoke about grief, homelessness, trauma, their poor mental health and other problematic substance use.

Many illustrated the impact that disruption had on attempts to quit smoking. As one participant explained:

“Well yeah I was homeless and that's pretty depressing…Hard to quit smoking… but I've been on and off homeless for about 4 years since my mother passed away.”

(*Intervention participant)*

Others expressed the connection between the chaos of life and relapse to smoking.

“I had a quit date in November and I was doing really well, I quit for probably a couple of weeks, and then I had a lot of stresses in my life, and I started just having one or two cigarettes again, and then at Christmas time I was very stressed and I started smoking a little bit more frequently… I was moving house, and I had to downsize, my partner died last year and there was a lot of things going on…It's very lonely without him.”

(*Intervention participant)*

Sometimes this impression of disruption reflected the powerlessness and lack of choice that comes from the poverty that was a common feature of peoples' lives. For some of our participants, smoking cigarettes meant they could not afford necessities.

“Exactly that's right, because it's easier to get by without food if you've got cigarettes, but getting by without cigarettes for the food, that's no fun, that's not a thing we're going to do.”

(*Intervention participant)*

There was a sense of isolation and stigma that connected financial difficulties and being a smoker.

“Yeah that's right. It's easy for the non-smokers to go—“*Oh the smokers are lepers anyway and who cares how expensive it is*”—you know, like if they just end up dying that's going to do everybody a favour.”

(*Intervention participant)*

In the context of these disrupted lives, smoking could be a distraction, a friend, a conduit for social connection, or a pleasurable pastime during mental health difficulties.

“I sort of find it's like a friend in a way you know it's time just to chill out and focus on something different and not what is at hand, you know, like not to be concerned with what I'm doing that particular day. I sort of think “I'll have a cigarette, bugger that” and just drift off a bit, …I don't have to worry about things.”

(*Intervention participant)*

Other substance use issues also intersected with smoking for some participants. For example, one participant stated:

“… and I think the first time that I actually started smoking cigarettes regularly was as a quitting mechanism for the cannabis.”

(*Control participant)*

***Subtheme: Quitting histories driven by persistence and desire***

Our discussions highlighted determination and desire to quit despite the challenges outlined above. In the context of difficult circumstances, most participants stated they had made multiple quit attempts previously.

“Made an attempt to quit? Yeah plenty of times.”

(*Control participant)*

Many had previously used a variety of methods to try to quit including both “cold turkey” and NRT.

“I've tried, I went to my doctor and we tried patches…Yeah tried like the gum and the mist and stuff like that.”

(*Control participant)*

For a small number of participants from both intervention and control groups, reflecting on these previous attempts to quit offered a hopeful perspective that they were now on a journey to successful quitting.

“No, like I said this is my 50th thousandth time I've tried, so yeah, I don't know, I guess for me it's just about not being disheartened every time I, like every time I quit I'm one step closer to quitting for good.”

(*Control participant)*

**Theme 2: Factors that participants perceive to impact quitting smoking**

Participants discussed a range of factors perceived to influence the success of quitting smoking including the desire to quit, their confidence and self-esteem, barriers and facilitators to change, and reasons for quitting.

***Subtheme: Desire to quit***

Many control and intervention participants spoke about the importance of having a desire to quit and demonstrate “self-control” to engage with tobacco treatment.

“But at the end of the day it's up to you, if you want to do something you have to make it happen for yourself, you know like you've got to—you can get outside support or help, but you've got to do it, you have to want to do it yourself.”

*(Control participant)*

Some participants took this idea further in talking about the need for that desire to be solidified and committed to. For some participants, this “want” to quit was linked to ideas around the requirement of an internal or mental shift about being “done” with smoking.

“Yeah I think because everyone's so different you know and some people might be like sick to death of smoking … and the health hazards and stuff like that whereas I am not overly concerned, like sometimes I think ‘Oh well, I am just going to end up dying from smoking and I am going to get cancer from it', but all those things go through your mind sometimes but I am not at the point … where I just hate it and I want to stop.”

*(Intervention participant)*

It appears that participants often believed that wanting to quit was essential to successfully ceasing smoking and that this had to happen within the right conditions. For example, one participant spoke of the importance of timing:

“Yeah it was just sort of at the right time that I sort of sought to get the support to help me and yeah it was just all timing. Probably if I had attempted to do it six months earlier it might not have worked or it might have taken a lot longer.”

*(Control participant)*

Some people described a lack of desire to quit smoking as being linked to living with their mental health challenges, including the will to live, and not feeling deserving of a better, healthy life.

“I mean I think that definitely in the past you know probably being depressed hasn't helped because there's sort of like a fatalistic …[and] ‘I don't even deserve to be healthy' sort of vibe going with that.”

*(Control participant)*

***Subtheme: Barriers and facilitators***

Unsurprisingly, numerous barriers and facilitators for quitting smoking were raised. These could be described as encompassing the personal (stress, mental health, boredom) and intersecting social domains (family, friends, living situation, environment, financial resources, and COVID-19).

*Personal*

Many participants spoke about smoking being interlinked with stress including as a coping response. Participants identified smoking as a reaction to daily stressors, mental health difficulties and sometimes boredom, particularly in the absence of other strategies for responding to these challenges.

“So, in the past it's been very difficult for me to stop because I get stressed out with things happening in life that I need stress relief, and I thought the cigarettes were actually helping me.”

*(Intervention participant)*

Stress was also described as a major trigger for relapse.

“I had a stressful thing happen and I started smoking again you know.”

*(Control participant)*

Most participants spoke about mental ill-health as a barrier to quitting. Smoking was described as a way to cope with poor mental health.

“Yeah, because I find that mental illness and smoking go hand in hand …. I know I've used it as a crutch to deal with my mental illness and in some ways it has helped to get through the tough times, even though it was causing me such great harm, but at the same time it did help me cope.”

*(Control participant)*

Severity of mental ill-health was seen as a barrier to quitting. As one participant stated:

“Yeah, I think if someone's really crook with mental health issues and they've only just been diagnosed it's going to be harder for them to stop than anyone else.”

*(Intervention participant)*

Others discussed that deterioration in their mental health could serve as a trigger for relapse to smoking.

“I have triggers if something goes wrong in my life, I start thinking about cigarettes again when things aren't going so good. So, if I start getting depressed or something goes wrong, I'll think I really do need a cigarette you know, that's usually how I've broken and ended up back on them.”

*(Intervention participant)*

Several participants commented that to be successful in cessation, mental health must also be addressed.

“When you're stressed and everything it's not a good time, you've got to address your mental health.”

*(Intervention participant)*

*Social*

Some participants referred to friends, family or partners who smoked when discussing barriers to cessation.

“I had friends that smoked too so that was a bit difficult obviously being around them and them smoking.”

*(Control participant)*

One participant described smoking as almost inevitable in the context of family history.

“My brother, he doesn't even try to give up. Really, I think he sort of settles for the fact that he's a smoker and my dad was a smoker all his life as well, so it runs in the family and both of my sisters are smokers, but they managed to quit maybe five, six, seven years ago. They both gave up but the males in the family they don't have such luck.”

*(Intervention participant)*

A small number of participants also described friends, family, or partners as facilitators to quitting, although one participant highlighted the fragility of this support.

“And my partner he smokes, so—he's supportive but also you know if he's having a cigarette, he's not going to go ‘Oh, you can't have one'.”

*(Intervention participant)*

The lack of a safe, supportive environment for cessation was described as a barrier.

“Yeah definitely, no, it's not a supportive and safe enough environment for me to be comfortable to be vulnerable and to fail, and I need that space before I can think about doing it.”

*(Control participant)*

Developing this theme, a small number of participants spoke about a change in environment as conducive to quitting. Specifically, their statements reflected the impact of escaping stressful or negative environments.

“Yeah, there were people, they were smoking cigarettes, or they were smoking other substances as well, and I've got them out of my life completely, and because that's why I wanted to move home, it wasn't a nice environment, it was too expensive, and I needed to be around people who wanted (inaudible). And I've moved by myself, and I'll be getting somebody else to move in, but hopefully I'll be requesting that they're a non-smoker, you know.”

*(Intervention participant)*

About half of our participants spoke about the impact of COVID-19. These participants described how the boredom and isolation associated with the pandemic made quitting harder.

“I think COVID-19 for me made it harder because I was stuck inside, I wasn't able to do my normal social things, so I was stuck at home, I was doing a lot of study on the computer, and I felt like I couldn't do anything else, so I thought ‘I'll have a cigarette'.”

*(Control participant)*

Only one participant described COVID-19 as a facilitator, stating that it made them more determined.

“It has been harder, but it's just made my resolve stronger.”

*(Intervention participant)*

The financial barrier to quitting centered on the challenge in getting started which potentially requires purchasing NRT products and cigarettes simultaneously and not having the money to do both.

“Yeah that's a barrier maybe for a lot of people you know—especially in my position I'm on a pension and stuff like that—the cost even though…I know that you can get all the products through the doctor, all the different sort of products and you know I was in the chemist the other day and noticed the price of them, I thought ‘Oh my god, there was no way I would have bought any of that!', I just literally would not have been able to afford to do that.”

*(Intervention participant)*

***Subtheme: Reasons to quit and benefits***

Reasons for attempting to stop or to cut down included physical health and financial benefits.

“The money I saved in the time I started, since I've really (inaudible) smoking now but I also appreciate the money I saved, that I spent on cigarettes before.”

*(Intervention participant)*

Two participants linked motivation to their children.

“And you know I'm driven by that motivation to quit, because I want to be a good role model for them.”

*(Intervention participant)*

**Theme 3: The benefits of a tailored approach for people with mental ill-health**

As part of the study, all participants were invited into the study, completed an initial assessment and peer researchers provided brief advice and written materials on stopping smoking. Across control and intervention arms, participants valued the components of the tailored approach that they were exposed to. There were varied experiences of difficulty in quitting and different levels of success.

***Subtheme: An invitation to quit***

Those who expressed gratitude for an invitation and the opportunity to participate in the “Quitlink” study were exclusively recruited to the trial *via* the direct mail postcards strategy. For some it appeared that they had minimal opportunities in the past to be involved in a tailored offer of support.

“If you hadn't made contact, I wouldn't have known to ring up and ask for help. Yeah, I feel very lucky to be pulled out of the hat and given a chance you know.”

*(Intervention participant)*

***Subtheme: Assessments as motivating and clarifying***

A number of control participants spoke about the study assessments as clarifying and motivating.

“Oh, it sort of made me…think about how much I was smoking and how much I'm spending and what I'm missing out on…Oh yeah because I spend all my money on cigarettes…Oh well it really makes me think about and consider having another attempt at getting them out of my, giving up smoking you know.”

*(Control participant)*

***Subtheme: The value of lived experience***

Some did not recall or distinguish the involvement of peer researchers who undertook recruitment, baseline assessment data collection provision of brief advice and written materials, from the research assistants who subsequently collected follow-up data *via* telephone. However, for those who valued peer researcher involvement, the importance of a unique shared understanding and experience of mental ill-health and smoking and recovery is reflected.

“I loved that she had you know that she was a peer researcher, that she had the experience of smoking and of mental health, and you know the impacts that they have together. So really just, you know, talking to (peer researcher) and knowing that she had that same experience and that kind of thing, was really helpful.”

*(Control participant)*

***Subtheme: Experienced support from Quitline counselors***

Participants identified a range of important study components that reflected an appreciation of relevant and experienced, continuous support.

The studies' deliberate use of experienced Quitline counselors (for the intervention group) was particularly appreciated. Several participants spoke about the helpfulness of Quitline counselor information to do with NRT use.

“I think the most helpful thing was having the phone calls with the Quitlink person or the—who was able to, you know, answer my questions how to use the products and also just helping me clarify my own thinking.”

*(Intervention participant)*

Others valued the skills and strategies offered by Quitline including recognizing and managing cravings, setting a plan, and creating specific and feasible goals.

“Yeah, the support he's given has been good. And just like being able to recognize going ‘Oh, I'm just having a craving right now, I don't need a cigarette'……Yeah, much more manageable having it kind of set out in a little, like you know, and he'd go ‘Are you comfortable with that?', you know, it was never kind of forced or—but I've kind of felt like yeah I can do that... So that I've found the most helpful, like getting little mini goals and achieving them and feeling good about achieving them.”

*(Intervention participant)*

Beyond information and skill building, participants valued Quitline counselors' support in meeting people “where they were at” and their continued encouragement. Furthermore, participants emphasized gratitude for a collaborative model of working.

“The goals that we set together, (inaudible) to achieve before the next phone call…Like the first one I think was ‘don't have a cigarette first thing in the morning, delay it'. So and I didn't think I could do that, so she talked me through it and like the possible problems and just yeah helped me comprehend that I could do it. And yeah, I did do it, that was the first goal.”

*(Intervention participant)*

Appreciation of the proactive referral and dedicated counselor approach that was employed in the intervention condition was highlighted.

“And yeah, not relying on a person to call the quitline off their own back, you know like have people asking the question like—but then you don't want to nag I guess, because that might, people might get resentful at that. But you know just be like ‘Oh hey, are you comfortable with this, or…'?”

*(Intervention participant)*

Four intervention participants spoke about the dedicated counselor and the increased appreciation of the intervention this brought.

“The quitline was brilliant…I had the same counselor every time, and she would call me, and she was wonderful, she had like great ideas, she was on point with where I was at…like we'd set a goal together and then I'd report back to her in a week's time, and it was really good.”

*(Intervention participant)*

One control participant's suggestion for proactive referral to Quitline (where the Quitline contacts the participant, which was offered to intervention participants only) further highlighted the value of having a dedicated counselor for each person who could offer continuity of care.

“It could be helpful to have that ongoing contact and … what would be important about that is maybe making … sure that the person feels like they don't have to retell their story, or they've been, you know you're working on I guess maybe what's happened in the last week and why or why not you weren't successful kind of specific things rather than the same message?”

*(Control participant)*

One intervention participant described the benefit of this relationship building to their progress.

“I think talking to the same person every time has been good, because he can kind of work on my progress with me and you know get to know me a little bit and—…I think if I just rung up and got a different person every time I spoke with them, it would be a bit weird. I think it's been good that I get to speak to the same person.”

*(Intervention participant)*

Further to the finding that some participants would not have actively sought tobacco treatment without the study invitation, a number of participants spoke about the fact that they also would not have engaged with Quitline if not for the study.

“…until I got the postcard about the study, I would never, I don't think I would've ever rung up and gone ‘Oh hi, I want to quit', like… I don't know I think maybe…for me I don't feel like smoking is like a big problem, I feel like maybe it would be …something I'd go to as a last resort, like if I had to quit smoking for health reasons... I think ringing up a place like that would kind of be like admitting defeat where I didn't want to admit defeat.”

*(Intervention participant)*

A couple of participants had trouble with access/availability of Quitline, although others found the opposite.

“Once I tried to ring in and the complaint I've got about that is I couldn't get through to anyone. I was really, really, desperate, and I rang the numbers, and I couldn't get through.”

*(Intervention participant)*

“I rang her yesterday because tomorrow I've got my quit date, but…she was supposed to be calling me in the morning, and I rang her just to reschedule the appointment, and she rang me straight back and we worked it out so that I can do both.”

*(Intervention participant)*

For those who did not engage with Quitline, this was due to not feeling comfortable with telephone, not feeling as though they needed that form of support; or as one participant described, a perception that the counselors could not be helpful without lived experience of smoking and quitting.

“I don't agree with talking with someone that's never smoked—I find that frustration because how can you honestly say relate to them in that way in regards—well you've never had a cigarette how can you counsel me if it's something you've never done.”

*(Intervention participant)*

***Subtheme: Practical resources***

Participants valued the physical resources that were provided: Quit brochures (both control and intervention) and NRT (intervention only). For a small number of control participants, the Quit information resources prompted a call to Quitline.

“Oh yeah, all the resources helped a lot because I never thought about even using Quitline and I ended up calling them and getting their support too.”

*(Control participant)*

However, for most control participants, these resources were not sufficient to prompt them to engage with Quitline. Intervention participants discussed the helpfulness of being provided free NRT.

“Yeah, well it was really good to get the parcel that had all the goods in it, because I wouldn't have been able to afford them on my income, I'm on a disability support pension… it was cheaper to go and buy a packet of cigarettes than it is to buy the nicotine patches and the spray and the lozenges, and the inhalers. So, I was really grateful to receive that.”

*(Intervention participant)*

Beyond their appreciation, participants said NRT was useful for managing cravings.

“I tried one day just wearing a patch just to see what would happen, but I found that I still craved a cigarette, so I thought ‘Ooh they're not going to work', but then with this study if you're having the NRT products—or the other ones the inhaler and the vaporiser I've found that was enough just to get me through that moment when I really wanted a cigarette I had something else I could do.”

*(Intervention participant)*

A minority of intervention participants were not motivated to use the NRT products, didn't find them helpful or had side effects.

“But for me, yeah, I didn't, and like I said I don't know why, but I didn't want to use them.”

*(Intervention participant)*

Similarly, a small number of both control and intervention participants spoke about using vaping as an additional product (not provided by the study intervention) during their study participation, to help them quit.

***Subtheme: Varying difficulty and success in quitting***

Within the theme of the benefits of a tailored approach sits ideas about the difficulty of quitting or cutting down as well as the varied levels of success experienced. Across control and intervention groups, some participants found quitting or cutting down difficult and frustrating.

“It is difficult…. You've got to change your whole kind of like way of doing things and thinking and being and stuff yeah, which is a lot you know.”

*(Intervention participant)*

However, a control participant who had made independent contact with Quitline noted:

“Look it's been frustrating; it's been very rewarding as well.”

*(Control participant)*

Some intervention participants that reported having quit for a length of time noted that they did not find it as difficult as they had imagined, even in the context of later relapsing back to smoking.

“Well, I think if someone had told me that it's—that quitting with support this way is relatively easy and certainly not as hard as I thought, I think that's the message, it's not as you think, I would have found that reassuring and more attractive.”

*(Intervention participant)*

The varied tobacco use recovery outcomes that participants spoke about highlighted the challenges of quitting but also confirmed that many were prepared to try.

***Subtheme: Instilling hope and building belief for future quit attempts***

Intervention participants spoke about next steps, trying again, working toward quitting and knowing they could do it now that reflected a sense of confidence and intent to try quitting again.

“In the past they (attempts to quit) weren't very good at all, but using the nicotine replacement products this time around and (inaudible) been a lot better, I know that I can quit now…It is, because…I honestly didn't think I could do it, and to have had just (inaudible) without a cigarette at all, that was amazing.”

*(Intervention participant)*

**Theme 4: A compassionate approach**

An overarching theme of compassion was identified. This encompassed ideas about the importance of health provider/service consideration of tobacco treatment for this population and the importance of their non-judgmental approach.

***Subtheme: Consideration***

There was mixed evidence for support from participants' current health professionals (psychiatrists, GPs, psychologists, support workers) during their study participation. Support received varied in quality and what impact it had on participants' experience of care. What was evident in those who had support and those who didn't, was that encouragement and compassion were key elements they appreciated.

“Yeah, they don't listen and like my doctors, for example, like I had to get stitches yesterday, and every time I see him it's give up cigarettes, give up cigarettes, it's like I just know he's going to say it, and I don't feel there's much compassion there.”

*(Intervention participant)*

“I mean I didn't really, I haven't really spoken to my psychologist about smoking, I mean when I first started seeing him, we did talk a lot about my alcohol consumption, and he did a little survey that they had for that. So, I don't know maybe—I actually have never had a psychologist ask me “Do you want to reduce your smoking?”

*(Intervention participant)*

This mixed support from health professionals was in marked contrast to the culture of compassion that participants generally described in our study across both control and intervention arms.

“Yeah, yeah look (Quitline counsellor)…but she was even beautiful. But I mean I was crying, like talking to them…opened up a lot about my mental health, and they were very compassionate.”

*(Intervention participant)*

The importance of encouragement was further highlighted by comments about support from others, e.g., friends, family.

“They encouraged me, you know my family and social worker I had at the time…That it was a very hard thing that I was doing, and it's like giving up something really big in your life, you know, something that played a big part in your life that wasn't there anymore, they were helping me get through that you know.”

*(Control participant)*

One participant spoke about the lack of consideration and compassion at a societal level, for people with mental ill-health.

“And I'm really glad that someone is taking an interest because the impression that I get…and the impression that we tend to get within our culture is that we are disposable, that we are disposable people … there is less interest in giving a shit basically.”

*(Intervention participant)*

***Subtheme: Connection***

Both control and intervention participants spoke about the study as providing an understanding point of contact and connection in their efforts to quit smoking.

“People think I should be able to just quit cold turkey and—whereas the people in the study …understand what's going on, have a little bit less judgement…I've got a good (inaudible) got a bad week because I've got a mental health diagnosis and I'm trying to do the best that I can you know… I've been grateful for someone being at the other end of the phone and it's really helped.”

*(Intervention participant)*

The importance of this was underlined by some control participants reporting they would have liked more contact.

“Just to have that reminder…because then I've fallen off the horse and then all of a sudden in 2 months' time I'm like ‘Oh yeah, shit that's what I wanted to do'.”

*(Control participant)*

***Subtheme: Non-judgmental approach***

In the context of a compassionate approach, the appreciation of a non-judgmental stance was specific to intervention participants and discussions about the relationship with Quitline counselors. Participants reported feeling safe in the knowledge that the counselors would not judge them for smoking relapse and in fact help them find learnings from this.

“But the person that I've been speaking with is very understanding, like even if I say “Oh I smoked a packet today”, he'll go ‘Okay what can we learn from that?', you know.”

*(Intervention participant)*

***Subtheme: Engagement and support***

In addition to the importance of the dedicated counselor (described in Theme 3) which was a component of the “Quitlink” intervention, an emphasis was placed on the counselors themselves and how essential it was for them to be consistently positive and supportive, so that participants could feel engaged in the relationship.

“…and you know she's just been a real support. She's actually like an angel, she really has been a supporter to me you know.”

*(Intervention participant)*

## Discussion

The present study explored participant experiences of a tobacco treatment trial for people who live with SMI, through interviews of participants assigned to either the active control or intervention conditions. The findings underscore the enormity of the challenges that our targeted population face and the considerations in providing support for them. They suggest that tailored tobacco treatment such as “Quitlink” has the potential to assist people on a journey to quitting, that often includes small gains, and that the multi-component interventions that include free combination NRT and evidence-based, compassionate support demonstrating a recovery-oriented approach are highly valued elements.

One of the most important findings was the appreciation from participants recruited exclusively *via* direct mail postcards of simply the invitation to join a tobacco treatment study. It is well established that the offer of tobacco treatment for people who experience SMI is suboptimal (8–10). This discrimination in provision of care and discrepancy in access is further evident in some of our intervention participants' expressions of feeling fortunate to be offered the invitation to engage with Quitline counseling and NRT.

Although all our participants were engaged with mental health service provision, and many also mentioned other primary care providers, it appears that most had not previously engaged with tobacco treatment or what had been offered to them was not person-centered and seemed ineffectual. This is despite other indicators that they were willing to engage with quitting. Our direct mail postcard approach yielded the greatest number of participants to the main “Quitlink” trial (31) and suggests utility in engagement of people who are often isolated and are at particular risk of being excluded from proactive support to cease smoking.

This finding adds to the evidence for the effectiveness of direct mail recruitment strategies found for other populations and interventions (34–36). Beyond being relatively low cost and low resource intensive (24), our participant perspectives on the value of invitation enrich our understanding of why this strategy can be successful. It may be that this approach bypassed the “gatekeeping” that can be a feature of recruitment that relies on staff to approach potential participants (37). Future tobacco treatment trials and mental health services should consider how direct mail recruitment strategies can be deployed to enable engagement and participation.

Most participants—across control and intervention arms—reported a general appreciation for the support offered by the study. Some participants appreciated being asked (during study baseline and follow up assessments) in an in-depth way about their smoking and found this clarifying and motivating; this was particularly so for the control participants. It may be that for control participants, who did not receive the “Quitlink” intervention, these research study assessments were helpful in supporting participants to understand their smoking behaviors as well as demonstrating consideration and “interest” in supporting people who experience SMI to quit smoking. People who smoke and experience SMI are not routinely asked about or provided with smoking cessation assistance and encouragement to quit from health care providers (38, 39). One of the barriers to this is the lack of knowledge reported by mental health staff about tobacco dependence and potential relationships between smoking and mental ill-health (40). Our findings highlight the value that people who smoke with SMI find in being asked about their smoking and the importance of continued efforts to upskill health professionals to engage in this conversation. This may include education and promotion of the effectiveness of quitlines and reminders to refer consumers.

Participants also valued the input of people with lived experience which is reflective of the growing evidence base for the role of peer support for people with mental ill-health (41). This role could be expanded in future tobacco treatment research and intervention efforts. This also relates to the value of a recovery-oriented approach to smoking cessation. Prior research has identified that recovery-oriented practice training in community mental health services positively impacted the process of personal recovery for consumers (42).

Predictably, the perceived value of the study and engagement with supports appeared greater for participants randomized to the intervention condition. This is consistent with the significantly more intensive support provided to this group. Many of the components of the Quitline counseling that intervention participants reported as helpful (recognizing and managing cravings, setting a plan and creating specific and feasible goals) are central to the quitline cognitive behavior therapy-based approach (43).

Most participants from the intervention arm reported that they would not have engaged with Quitline if they were not involved in the trial, hence demonstrating the value and importance of proactive approaches to tobacco treatment for people living with SMI. Routine assessment of smoking and provision of brief advice that proactively links people to underutilized best practice treatments such as Quitline and combination NRT has huge potential to improve the quality of life for people experiencing mental illness who smoke but it remains an ongoing challenge for both health and mental health services. Relying on spontaneous use of tobacco treatments means that many will miss out on valuable treatment that they need and deserve. The success evident in the current study in engaging and connecting people who experience SMI and smoke with quitline is an important outcome in terms of demonstrating that it is an acceptable and valued service.

Beyond the need for increased engagement with tobacco treatment, the findings of the current study suggest tailored and relevant intervention for this population is crucial. This is evident in participant appreciation for peer researchers and Quitline counselors experienced in supporting people experiencing SMI to stop smoking. The counselors appeared to support not only cessation behaviors but also elements of recovery, for example, hope and respect (44). Future trials should include a recovery measure to assess this more formally.

One of the key findings that arose from the qualitative analysis of participant interviews is the importance that participants placed on the compassionate approach they perceived the study to offer. Although a compassionate approach was not a key component of the original trial or intervention design, it seems that this style was initially established by our peer researchers and followed through by quitline counselors (for those allocated to the intervention) as well as by the follow-up assessors. Therefore, whilst evidence-based interventions such as quitline and NRT are critical to tobacco treatment, the spirit of delivery is also highly valued. Our participants were attuned to the experience of feeling judged and not being heard, a frequent experience of past interventions. This suggests that, in addition to smoking cessation expertise, tobacco treatment services, should continue to employ professional counselors and highlight the importance of foundational counseling skills including openness, a non-judgmental stance, and compassion, that are aligned with a recovery-oriented approach for clients experiencing SMI (45). This finding also adds to the value of quitline services for this population and future efforts should include highlighting this evidence-based but also supportive approach to increase linkage and engagement between mental health services, consumers and quitlines. The extra training that quitline counselors received for this project in tailoring their intervention for people experiencing SMI likely added to the value that participants found in the support. Around one third of quitline users in Victoria Australia report mental-ill-health and it is closer to a half of U.S. quitline clients (13) thus ongoing professional development for quitline counselors in assisting clients experiencing mental ill-health remains a key priority area for both training and individual and group supervision.

The intervention offered in this trial was designed with consideration for the difficulties faced and additional support required for smokers experiencing SMI. However, our interviews illuminated the picture of the significant life challenges of our participants and reflected the entrenched social, financial, and psychological disadvantages common to this population (25). Smoking and multiple previous attempts to quit were intertwined with these disrupted lives and depicted a long and complex history for our participants before entering the trial.

The barriers and facilitators to tobacco use recovery raised by participants continued the thread of disrupted lives that ran through the interviews. Whilst there were components within the psychological and social milieu that sometimes worked for or against participants' engagement with tobacco treatment, there was an overall sense of competing imperatives that made it extremely difficult to make quitting smoking a priority. The complex life stressors often endured by people who experience SMI was the main barrier reported by our participants. Considering this, the resources required to pursue a quitting journey while balancing these complexities and potential fluctuations in an individual's mental health, and the erosion of self-efficacy from repeated failures to quit smoking (46) poses a significant challenge. A small number of participants spoke about using electronic cigarettes as an additional product they used to support their attempts to quit smoking. Future research among people experiencing SMI is needed to monitor how e-cigarettes are being used, their impact on mental health and their effectiveness for smoking cessation.

Many participants felt that both a desire and a solid commitment to quitting are crucial for tobacco use recovery. Descriptions of this were somewhat intangible. This is reflective of findings with smokers from the general population that have demonstrated a belief that an unambivalent desire to quit is a necessary condition for successful tobacco use recovery (47). It is important to consider myths around motivation when engaging people in tobacco treatment, including that, when making behavior change, ambivalence is normal (48), motivation may fluctuate, relapse is common, and that a commitment to change can be made in spite of this. This may be particularly important for people who experience SMI, where self-doubt is prevalent, feeling vulnerable may be difficult, and the possibility of failure feels perilous and disheartening (49). The notion of an environment conducive to quitting was described as a potential facilitator, which fleshed out the idea that in addition to the desire to quit smoking, participants felt a supportive environment where it was safe to fail was important. In the context of disrupted lives, there are often systemic barriers to cessation, for example, during in-patient hospitalization (50) and supported residential facilities (51). Even though these are now often smoke free, our participants suggested congregate housing settings and stressful living environments can disrupt tobacco use recovery efforts.

Despite the numerous barriers, many participants did speak about making quit attempts. This contrasts with attitudes that people with mental illness are not interested in quitting (52). Our participants' comments supported previous findings that people with mental illness are highly motivated to stop smoking (5). Further, some participants assigned to the Quitlink intervention, who successfully quit, found that with support, tobacco use recovery was not as challenging as they imagined. The benefits of trial participation went beyond the immediate; including instilling hope, confidence and building belief to try quitting again in the future. This is an important finding; whilst we need to acknowledge the challenges for this population and how these impact quitting, as researchers and service providers we need to be cautious that in doing so, the message is not communicated to either consumers or health professionals that quitting smoking for people experiencing SMI is so difficult that it is not worth attempting or may endanger their mental health (53). Health messaging should be non-discriminatory and hopeful, challenging low expectations. The barriers are not insurmountable and quitting with the help of tailored and evidence-based support is possible.

## Strengths and limitations

Due to the challenges with recruitment to the main trial (31), a convenience sampling method replaced the intended purposive sampling strategy for the current study. Delays in recruitment for this qualitative study led to participants that were recruited in the main trial *via* the face-to-face initial recruitment strategy being not represented in these 2-month interviews. These participants were largely from supported living services and likely to have experienced greater mental ill-health symptoms (than subsequent recruits who were recruited *via* direct mail postcard or online). There will be opportunities to explore their experience in the 5- and 8-month interviews and new themes may be identified. However, participants from both control and intervention arms of the trial took part in our interviews and the sample characteristics were comparable to that of the overall trial population (see Table 1).

The participants in the current study reflect the views of those who agreed to participate in a tobacco treatment trial and subsequently to share their experiences in an interview. Consequently, the findings may not generalize to those with lower levels of engagement with the study. However, our findings offer insight into a group of participants who did not necessarily want to quit smoking but were interested in or open to being offered tobacco treatment support options. Peer researchers were involved in all aspects of the current study including recruitment, data collection and analysis. Their lived experience was one of the key perspectives guiding this research and adds to the value and uniqueness of our findings (54, 55).

## Conclusions

Although people who experience SMI face unique difficulties in quitting smoking, our findings support previous evidence that people who experience mental illness are often highly motivated to stop smoking (5) and can engage with tobacco treatment when support is appropriate and tailored (56, 57). Our findings add nuance to this evidence in demonstrating that people who experience SMI highly value health professionals and services (e.g., peers and quitline counselors) who understand the complexities of mental ill-health and intersecting challenges and employ a compassionate approach. Our findings suggest that in addition to the importance of tobacco treatment services and programs employing evidence-based and tailored support for people who experience SMI, there is a clear need for a recovery orientation.

## Data availability statement

The datasets presented in this article are not readily available because this would limit participant confidentiality. Requests to access the datasets should be directed to kristen.mccarter@newcastle.edu.au.

## Ethics statement

The study was reviewed and approved by St Vincent's Hospital Melbourne HREC, Cancer Council Victoria HREC, and University of Newcastle HREC. The patients/participants provided their written informed consent to participate in this study.

## Author contributions

The first draft of the paper was written by KM with significant input from LB, MM, NC, CB, and LH, followed by the remaining authors. All authors contributed to manuscript revision, read, and approved the submitted version.

## Funding

This project was funded by the National Health and Medical Research Council (APP1139125). The funder had no role in study design, data collection and analysis, decision to publish, or preparation of the manuscript. AB holds a NHMRC Fellowship (APP1135901) and Faculty of Health and Medicine, University of Newcastle, Gladys M Brawn Fellowship.

## Conflict of interest

The authors declare that the research was conducted in the absence of any commercial or financial relationships that could be construed as a potential conflict of interest.

## Publisher's note

All claims expressed in this article are solely those of the authors and do not necessarily represent those of their affiliated organizations, or those of the publisher, the editors and the reviewers. Any product that may be evaluated in this article, or claim that may be made by its manufacturer, is not guaranteed or endorsed by the publisher.
