# Supplementary material for: The value of compassionate support to address smoking: A qualitative study with people who experience severe mental illness
Source: Front Psychiatry. 2022 Oct 6;13:868032. doi: 10.3389/fpsyt.2022.868032 (PMC9583161; doi:10.3389/fpsyt.2022.868032)
Supplement: Supplementary file 1 [file Table_1.DOCX]

**Quitlink qualitative interview questions**

These will be semi structured interviews, so this is only a guide to the interviews. The interviewer may explore some responses in more depth.

- What has been your experience of attempts to quit? Have you been successful? What do you think has been helpful or not helpful in your attempts to quit? What are the barriers you have faced in your attempts to quit (prompts – environment, support people, smoking culture, mental health issues)
- What has been your experience of getting help to quit?
- Have you had assistance from peer workers? Do you think your attempts to Quit were enhanced by involving peer workers? Why? How?
- Is there anything that could have been done differently to enhance the role of peer workers?
- What is your impression of the Quitline or other telephone counselling? (prompts – strengths, weaknesses, things you think could be done differently)
- What role did any telephone counselling or other support you have had play in your attempts to quit? Are there any other things that have been helpful (prompts – NRT, peer workers, engagement with mental health services and support network, peer worker follow up?). Please explain your thoughts on this. Do you think some people are more likely to respond positively to getting support to quit than others? Why? Could this be enhanced or improved? How well did any support you have had to quit address the barriers we discussed earlier? What could be done differently to assist you with the barriers to quitting for you?
- Do you have any other ideas about quitting smoking you would like to share?
